# Supplementary material for: Message framing to promote solar panels
Source: Nat Commun. 2023 Nov 8;14:7187. doi: 10.1038/s41467-023-42904-0 (PMC10632465; doi:10.1038/s41467-023-42904-0)
Supplement: Supplementary file 1 — Supplementary Information [file 41467_2023_42904_MOESM1_ESM.pdf]

Message framing to promote solar panels

Dominik Bär*[1], Stefan Feuerriegel[1], Ting Li[2], and Markus Weinmann[2,3]

[1]LMU Munich, Geschwister-Scholl-Platz 1, 80539 Munich, Germany

[2]Rotterdam School of Management, Erasmus University Rotterdam, Burgemeester Oudlaan 50, 3062 PA Rotterdam, Netherlands

[3]University of Cologne, Albertus-Magnus-Platz, 50923 Cologne, Germany

*Corresponding author: baer@lmu.de

Supplementary Materials

Contents

1 Background **3**

2 Solar energy in the Netherlands **7**

3 Presentation of interventions (field experiment) **9**

4 Regression results **11**

5 Robustness checks for the field experiment **12**

5.1 Alternative model specifications (probit, linear model) 12

5.2 Alternative model with intercept/reference condition 14

5.3 Subsetting the data to visitors from the Netherlands 16

5.4 Additional controls (device type, weekday) 17

5.5 Frequency of treatment arms 19

6 Regional heterogeneity in the commitment to solar panels **20**

7 Online experiment **25**

7.1 Survey for online experiment 33

1 Background

Message framing has been used in various settings to promote pro-environmental behavior. Yet, there is an ongoing scholarly discourse on whether messages targeting oneself or the environment are more effective. The reason is that the effectiveness of message framing may vary across goods and may thus be different for repeated or low-cost behaviors (e.g., choosing electricity tariffs, or reusable bags) as compared to large-scale investments. In the following, we provide a brief overview of different explanations for why messages targeting both oneself and the environment could potentially be effective in promoting solar panels among the broader public.

Message framing targeting oneself or the environment

Pro-environmental decision-making is often characterized by a social dilemma [1]. That is, individuals need to find a balance between contributing to the collective social good and maximizing their personal benefits [1]. To motivate pro-environmental behavior, message framing thus commonly draws attention to personal benefits or the environment by emphasizing positive environmental outcomes [1, 2, 3]. Existing works have studied social dilemmas for repeated or low-cost behavior, but not for decisions behind large-scale investments. For large-scale investments such as the adoption of solar panels, individuals are also facing such a dilemma: On the one hand, the large environmental impact contributes significantly to the collective social good. On the other hand, the large up-front costs mandate a careful financial evaluation where economic considerations may hold even greater importance as compared to repeated or low-cost behaviors. To this end, existing findings focusing on repeated or low-cost behaviors may not be directly applicable to the context of large-scale investments due to the large up-front costs. Therefore, it remains unclear whether messages designed to promote large-scale investments should be targeted at oneself or the environment.

A common assumption of message framing targeting oneself is that individuals' decisions are

driven by self-interest [1, 3, 4]. As such, interventions highlighting economic gains, such as additional earnings or cost savings, are considered effective for motivating pro-environmental behavior [1, 5]. Previous research on the effectiveness of message framing for repeated or low-cost behaviors found that targeting oneself is effective [4, 6, 7]. However, we study large-scale investments in the form of solar panels where decision-making processes are likely to be governed by the substantial up-front costs and time commitment [8, 9, 10, 11]. To this end, we expect economic considerations to be essential for the decision-making process around solar panels as a large-scale investment, irrespective of individuals' self-interest. In this sense, highlighting economic benefits would reduce the perceived financial risks of such investments or simply outweigh collective social gains [1].

On the contrary, targeting oneself, that is, maximizing individual benefits, often neglects people's desire to uphold a positive self-concept [3,12,13]. By emphasizing the environmental benefits and thus the collective social good of a decision, messages may appeal to a person's positive self-concept and motivate pro-environmental behavior. In fact, environmental preservation is important to a majority of the population and most people also express a willingness to act environmentally friendly [14, 15]. Consequently, previous research has found that environmental concerns are an important driver of human decision-making [3, 11, 16, 17, 18, 19, 20, 21, 22] and messages targeting the environment have been previously used to effectively motivate pro-environmental behaviors [2, 3, 16, 17, 18, 20, 23]. Hence, message framing targeting the environment may also be effective in promoting solar panels, yet it is unclear whether pro-environmental behavior is a dominant driver for large-scale investments due to their high up-front costs.

The role of personal values, social norms, and individual beliefs

The effectiveness of message framing may also relate to how messages appeal to personal values, social norms, and individual beliefs. For personal values, previous research has identified hedonic, egoistic, altruistic, and biospheric values as important drivers of pro-environmental behavior [24].

Here, hedonic and egoistic values tend to impede pro-environmental behavior while altruistic and biospheric values foster support for environmental policies [24]. In the context of solar panels, messages that highlight financial benefits may thus alleviate concerns surrounding the costs associated with pro-environmental behavior, thereby appealing to an individual's hedonic and egoistic values. In contrast, emphasizing environmental benefits may appeal to individuals' altruistic and biospheric values, hence increasing the rate of customers committing to solar panels.

In addition to personal values, social norms have been shown to be a strong driver of pro-environmental behavior [25, 26, 27]. Individuals may try to align their behavior with what they assume to be socially expected. Previous research has shown that peer effects are important drivers of solar panel installations [9, 28, 29, 30]. This may be because individuals seek to align with their peers to match social expectations (e. g., "my neighbors act environmentally friendly, so do I"). In the context of message framing, one would thus expect that environmental appeals are more effective compared to financial appeals as they are socially more desirable. However, the large up-front costs associated with solar panels may once again outweigh social norms.

Furthermore, individual beliefs may also influence investment decisions for solar panels. For example, people may have unreasonable beliefs with respect to solar panel adoption, e. g., by overestimating costs or amortization periods, thus leading to fewer customers committing to solar panels. In fact, previous research showed that wrong perceptions of the battery range of electric vehicles hindered the uptake of environmentally-friendly mobility [31]. However, providing additional information effectively addressed a range of such concerns and significantly increased the willingness to pay for electric vehicles [31]. Similarly, unreasonable beliefs on the adoption of solar panels such as overestimated costs and amortization periods may be alleviated by messages that highlight cost savings or additional earnings. As already stated above, highlighting economic benefits may reduce the perceived financial risks of such investments [1]. Taken together, this implies that messages that make the financial benefits of solar panels more salient may lead to higher rates of commitment.

The influence of personal and normative goals in decision-making

Message framing may also be guided by the concept of goal framing [32, 33, 34]. Goal framing posits that people's cognitive and motivational processes, as well as their subsequent actions, are influenced by the goals they are presently pursuing. These goals can be triggered by external cues, which make those goals more salient and temporarily give them weight, thereby making them a focal goal or goal frame. Such goals can become automatic reactions to cues even without deliberation [35]. Goal framing targets three overarching goals: hedonic goals (aimed at immediate emotional well-being), normative goals (acting in accordance with group expectations), and gain goals (protecting and improving one's resources). In this work, we focus on gain goals (individual benefit) versus normative goals (environmental benefits), thereby relating to the above discussion on whether economic cues or pro-social behavior exert stronger motivations to adopt solar panels.

In conclusion, the effectiveness of message framing to promote solar panels may depend on various factors. Our study employs different messaging strategies, which target either oneself or the environment, to test different motivations for pro-environmental behavior. Due to the high up-front costs of solar panels, existing findings from repeated or low-cost behaviors may not be transferable which thus requires new research as in our work. By targeting oneself and emphasizing cost savings or additional earnings, we aim to appeal to economic considerations that are likely to influence customers' decisions and thus lead to a higher rate of commitment to solar panels. In contrast, by targeting the environment, we aim to highlight the prevention of environmental harm by reducing emissions or environmental benefits by producing green energy. Thereby, we test whether the adoption of solar panels can be motivated by appealing to an individual's positive self-concept or social norms. We test these opposing hypotheses in a large-scale field experiment to provide robust causal inference on which type of message framing is effective in promoting solar panels among the broader public.

2 Solar energy in the Netherlands

The participants of our field experiment are from the Netherlands and, hence, are all subject to the regulatory framework and financial incentives provided by the Dutch government and the European Union. In the following, we provide a brief background about solar panel adoption in the Netherlands and the regulatory framework.

The Netherlands is a leading producer of solar energy in Europe. In total, the installed solar capacity in the Netherlands amounts to 19,143 MW, which covers around 14 % of the country's total energy production in 2022 [36]. As such, the solar energy capacity per capita amounts to 1,044 W per capita and is thus the largest capacity per capita within the European Union [37]. For comparison, the solar energy capacity in the United States amounts to only 217 W per capita [38]. Overall, this underlines the importance of solar energy in the Netherlands. Nevertheless, it may also imply that the market for solar panels is already somewhat saturated, which, in turn, would imply that the interest in adopting solar panels could be greater in other parts of the world where the penetration of solar panels is low (which thus presents a challenging customer base for our experiment).

A significant share of solar panel capacity in the Netherlands originates in the residential sector. In 2022, around 46 % of solar capacity growth was added by the residential sector, which is more than by any other sector [37]. In fact, a majority of the Dutch population supports the transition towards green energy with 83 % specifically advocating for greater use of solar energy in the Netherlands [39]. Recently, an important driver for the strong growth of residential solar energy in the Netherlands was the Russian invasion of Ukraine which led to a major energy crisis and spiking energy prices across Europe [40]. The high energy prices improved the business case for residential solar panels and thus led to additional solar panel installations by private households [37]. Note, however, that our field experiment took place before the Russian invasion and thus before the energy crisis.

The regulatory framework in the Netherlands promotes the adoption of residential solar panels through multiple financial incentives. An important characteristic of solar energy generation in the Netherlands is the so-called net-metering. Net-metering allows households to obtain credit for supplying energy to the power grid that can later be used to offset the costs of energy consumption from the grid in times when a household's solar panels do not produce enough energy. Hence, households can significantly lower their energy bill by installing solar panels, especially, because net-metering is free of charge, and there are no limits on the amount of energy that can be supplied to the grid [37]. In the upcoming years, the system of net-metering will be phased out, and, instead, households will be directly compensated for the energy they produce [37]. In addition, the Dutch government provides direct financial subsidies of up to EUR $\approx$2,000 (depending on the size of the system) that support solar panel installations [41]. Of note, the financial subsidy is the same for all households. Households further profit from lower energy taxes due to the lower energy consumption from the grid [42]. Overall, there are multiple financial benefits to adopting solar panels for households in the Netherlands. In our field experiment, these are reflected by that message framing states expected financial savings of around EUR 813 per household, which is a conservative estimate that was set by our partner company (see Section 3 for details).

Our interventions are designed independent of our contextual setting that focuses on the Netherlands to ensure the widespread applicability of our findings. On top of that, regulatory frameworks similar to those in the Netherlands are implemented in vast parts of the European Union (e.g., Germany) and other regions of the world. Hence, our findings should also be transferable to other countries.

3 Presentation of interventions (field experiment)

In the field experiment, the interventions were presented to customers visiting the e-commerce website. The online retailer advertised PV systems using the term "solar panels" given the widespread use of the term among potential customers but sold and installed whole PV systems (i. e., including an inverter, mounting system, etc.).

On every page of the website, the interventions were displayed via a call-to-action box (see screenshots in Figure 1a–c). The call-to-action box contained the messages targeting either oneself or the environment. In addition, the messages framed the adoption of solar panels as cost savings versus earnings (oneself) or reducing emissions versus generating green energy (the environment). These messages varied according to the four interventions (i. e., *Self–Save*, *Self–Earn*, *Environment–*CO_2, and *Environment–Green*). For all interventions, the presentation also included information describing the installation process and a button to start the adoption form and commit to solar panels. If a visitor scrolls down, then the call-to-action box is replaced by a banner at the top (Figure 1d). This ensures that the interventions are always visible. For each customer, the intervention remained the same across all visits to the website by means of tracking.

The interventions showed a price effect of EUR 813 per year. The price was set by the online retailer and is a conservative estimate of the potential savings that should hold for the majority of solar panels installed in the Netherlands, regardless of the specific location and other housing characteristics. For comparison, a newly installed solar energy system that covers the average electricity consumption of 2.81 MWh [43] of a Dutch household in 2021 requires an initial investment of approximately EUR 4,000 [44]. The system incurs yearly operating costs of around 2 % [45] of the initial investment, resulting in a total cost of EUR 5,920 over 25 years. Assuming that this system saves EUR 1,049.73 per year, which is the average amount spent on electricity by a household in the Netherlands in 2021 [42], the total amount saved over 25 years would be EUR 20,323.15 or EUR 813 per year.

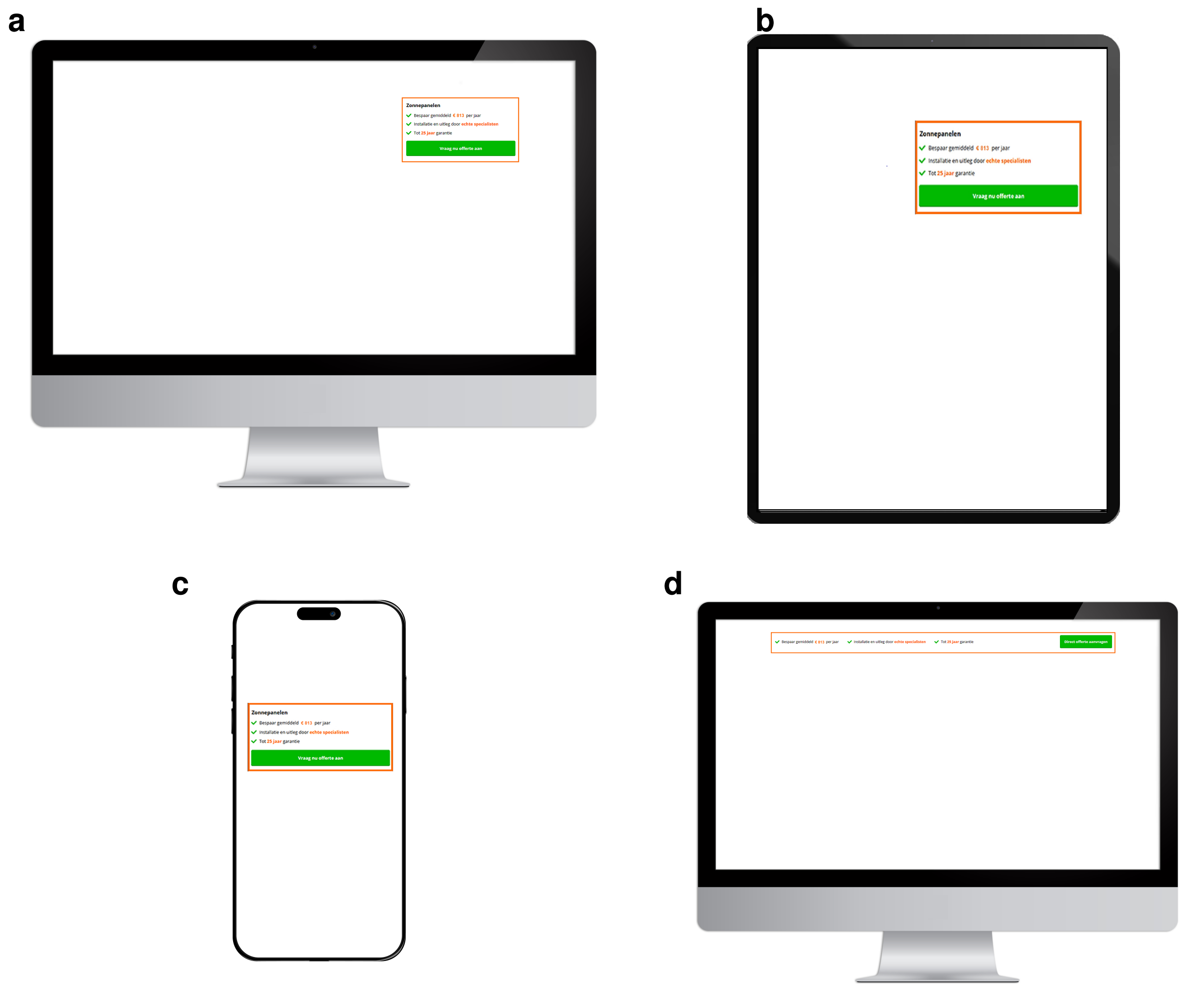

Supplementary Figure 1: Interventions as shown in the field experiment The presentation of the interventions is shown for different devices: **a**, desktop; **b**, tablet; and **c**, smartphone. Here, the text (e. g., "Save on average € 813 per year") varied according to one of the four interventions as specified in the Methods. Keywords referring to the target (i. e., oneself or the environment) and the framing in the presentation were also highlighted in orange during the online experiment. Note that a robustness check controlling for the different device types led to consistent findings (see Section 5). **d**, If a visitor scrolls down, the call-to-action box is replaced by a banner ensuring that the interventions are always shown on the screen. Due to confidentiality, the background is sanitized.

4 Regression results

Detailed results, including all estimated coefficients from the field experiment, are reported in Table 1. We find that framing the decision to adopt solar panels as cost savings for oneself yields the largest estimated effect. The estimated coefficient is larger than those for the other messages. This holds true for *Self–Earn*, *Environment–CO_2*, and *Environment–Green*. Note that coefficients may be negative on the log-odds scale, i. e., prior to applying the inverse-logit transformation (i. e., $e^{\alpha_{\text{Condition[i]}}}/(1 + e^{\alpha_{\text{Condition[i]}}})$). Hence, after applying the inverse-logit transformation, we see a positive rate of commitment, in line with our expectations.

Supplementary Table 1: Estimation results for the main analysis of the field experiment. The table shows the regression results for our logistic model from the main analysis. The first column reports the estimated coefficients on a log-odds scale ($\alpha_{\text{Condition[i]}}$) from the logistic regression. The last column (Rate of commitment) reports the corresponding estimated rate of commitment (i. e., $e^{\alpha_{\text{Condition[i]}}}/(1 + e^{\alpha_{\text{Condition[i]}}})$). Note that coefficients may be negative on the log-odds scale, i. e., prior to applying the inverse-logit transformation. P-values are calculated using tow-sided t-tests. Abbreviations: b = coefficient, s.e. = standard error, CI = confidence interval, AIC = Akaike information criterion, Obs. (N) = Number of observations.

	b	$s.e.$	P	95 % CI	Rate of commitment
Self–Save	−2.88	0.05	0.00	[−2.99; −2.78]	0.05
Self–Earn	−3.13	0.06	0.00	[−3.26; −3.02]	0.04
Environment–CO_2	−3.15	0.06	0.00	[−3.27; −3.03]	0.04
Environment–Green	−3.18	0.06	0.00	[−3.31; −3.06]	0.04
AIC	9698.56				
Obs. (N)	26873				

5 Robustness checks for the field experiment

Detailed estimation results for the robustness checks from the field experiment are in Table 2–Table 5. Note that the estimated coefficients may be negative depending on the scale. However, a larger coefficient implies a greater estimated rate of commitment.

5.1 Alternative model specifications (probit, linear model)

We evaluated the robustness of our results in the field experiment by repeating the analysis with different model specifications (i. e., probit model, linear model). The estimation results are in Table 2. Consistent with our findings in the main analysis, we found that the condition *Self–Save* has the largest estimated coefficient for all model specifications.

Supplementary Table 2: Estimation results for varying model specifications for the field experiment. The table shows the regression results for our logistic model (Logit), probit model (Probit), and linear probability model (Probit). P-values are calculated using tow-sided t-tests. Abbreviations: b = coefficient, s.e. = standard error, CI = confidence interval, AIC = Akaike information criterion, Obs. (N) = Number of observations.

	Logit				Probit				Linear model			
	b	$s.e.$	P	95 % CI	b	$s.e.$	P	95 % CI	b	$s.e.$	P	95 % CI
Self–Save	−2.88	0.05	0.00	[−2.99; −2.78]	−1.61	0.02	0.00	[−1.66; −1.57]	0.05	0.00	0.00	[0.05; 0.06]
Self–Earn	−3.13	0.06	0.00	[−3.26; −3.02]	−1.73	0.03	0.00	[−1.78; −1.68]	0.04	0.00	0.00	[0.04; 0.05]
Environment–CO_2	−3.15	0.06	0.00	[−3.27; −3.03]	−1.74	0.03	0.00	[−1.79; −1.68]	0.04	0.00	0.00	[0.04; 0.05]
Environment–Green	−3.18	0.06	0.00	[−3.31; −3.06]	−1.75	0.03	0.00	[−1.81; −1.70]	0.04	0.00	0.00	[0.03; 0.04]
AIC	9698.56				9698.56				−8860.72			
Obs. (N)	26873				26873				26873			

5.2 Alternative model with intercept/reference condition

We then checked the robustness of our results from the field experiment when including an intercept in the logistic model and varied the reference condition. This yielded four regression models in which each intervention acted once as a reference. The results are displayed in Table 3. Of note, the total effect of a condition is calculated by summing the coefficient of the intercept and the coefficient of the specific condition. Overall, we find that the condition *Self–Save* yields the largest coefficient in all models. Hence, the results from the main analysis are supported.

Supplementary Table 3: Estimation results for a logistic regression with varying reference categories in the field experiment. The table shows the regression results when *Self–Save* (a), *Self–Earn* (b), *Environment–CO_2* (c), and *Environment–Green* (d) serve as the reference category, respectively. P-values are calculated using tow-sided t-tests. Abbreviations: b = coefficient, s.e. = standard error, CI = confidence interval, AIC = Akaike information criterion, Obs. (N) = Number of observations.

	(a)				(b)				(c)				(d)			
	b	$s.e.$	P	95 % CI	b	$s.e.$	P	95 % CI	b	$s.e.$	P	95 % CI	b	$s.e.$	P	95 % CI
Intercept	−2.88	0.05	0.00	[−2.99; −2.78]	−3.13	0.06	0.00	[−3.26; −3.02]	−3.15	0.06	0.00	[−3.27; −3.03]	−3.18	0.06	0.00	[−3.31; −3.06]
Self–Save					0.26	0.08	0.00	[0.10; 0.41]	0.27	0.08	0.00	[0.11; 0.43]	0.30	0.08	0.00	[0.14; 0.47]
Self–Earn	−0.26	0.08	0.00	[−0.41; −0.10]					0.01	0.09	0.89	[−0.16; 0.18]	0.05	0.09	0.58	[−0.12; 0.22]
Environment–CO_2	−0.27	0.08	0.00	[−0.43; −0.11]	−0.01	0.09	0.89	[−0.18; 0.16]					0.04	0.09	0.68	[−0.14; 0.21]
Environment–Green	−0.30	0.08	0.00	[−0.47; −0.14]	−0.05	0.09	0.58	[−0.22; 0.12]	−0.04	0.09	0.68	[−0.21; 0.14]				
AIC	9698.56				9698.56				9698.56				9698.56			
Obs. (N)	26873				26873				26873				26873			

5.3 Subsetting the data to visitors from the Netherlands

We also repeated our main analysis from the field experiment only with data based on a subset of visitors with a location in the Netherlands. The results are in Table 4. Overall, we found that all coefficients are in good agreement with the results from our main model, hence supporting the robustness of our findings.

Supplementary Table 4: Robustness of results in the field experiment when accounting for the location of visitors which was inferred based on the IP address. The first model (Main analysis (all visitors)) represents the regression results from the main analysis, which did not exclude participants based on the location from which they accessed the website (e. g., it may include residents from other countries). The second model (Subset (Netherlands only)) repeats the analysis based on the subset of visitors with a location in the Netherlands (i. e., this excludes residents from other countries, but this may filter out Dutch visitors during travels abroad). P-values are calculated using tow-sided t-tests. Abbreviations: b = coefficient, s.e. = standard error, CI = confidence interval, AIC = Akaike information criterion, Obs. (N) = Number of observations.

	Main analysis (all visitors)				Subset (Netherlands only			
	b	$s.e.$	P	95 % CI	b	$s.e.$	P	95 % CI
Self–Save	−2.88	0.05	0.00	[−2.99; −2.78]	−2.69	0.05	0.00	[−2.80; −2.58]
Self–Earn	−3.13	0.06	0.00	[−3.26; −3.02]	−2.92	0.06	0.00	[−3.04; −2.80]
Environment–CO_2	−3.15	0.06	0.00	[−3.27; −3.03]	−2.93	0.06	0.00	[−3.06; −2.81]
Environment–Green	−3.18	0.06	0.00	[−3.31; −3.06]	−2.98	0.06	0.00	[−3.11; −2.86]
AIC	9698.56				8967.86			
Obs. (N)	26873				21472			

5.4 Additional controls (device type, weekday)

Finally, we included further controls in our logistic regression from the field experiment. We included fixed effects for the device type and the weekday a customer accessed the website from. The results are in Table 5. Consistent with our main findings, all coefficients are in good agreement, hence supporting the robustness of our results.

Supplementary Table 5: Estimation results for the main analysis in the field experiment, including further controls. The first model (Model 1) reports the regression results from the logistic regression without controls. The second model (Model 2) reports the regression results from the logistic regression including fixed effects for the device type. The third column (Model 3) reports the regression results from the logistic regression including fixed effects for weekdays. P-values are calculated using tow-sided t-tests. Abbreviations: b = coefficient, s.e. = standard error, CI = confidence interval, AIC = Akaike information criterion, Obs. (N) = Number of observations.

	Model 1				Model 2				Model 3			
	b	$s.e.$	P	95 % CI	b	$s.e.$	P	95% CI	b	$s.e.$	P	95 % CI
Self–Save	−2.88	0.05	0.00	[−2.99; −2.78]	−2.5	0.06	0.00	[−2.62; −2.39]	−2.94	0.09	0.00	[−3.12; −2.77]
Self–Earn	−3.13	0.06	0.00	[−3.26; −3.02]	−2.71	0.07	0.00	[−2.84; −2.58]	−3.19	0.09	0.00	[−3.38; −3.01]
Environment–CO_2	−3.15	0.06	0.00	[−3.27; −3.03]	−2.76	0.07	0.00	[−2.90; −2.63]	−3.21	0.09	0.00	[−3.40; −3.03]
Environment–Green	−3.18	0.06	0.00	[−3.31; −3.06]	−2.79	0.07	0.00	[−2.93; −2.66]	−3.25	0.09	0.00	[−3.43; −3.06]
Device type	NO				YES				NO			
Weekday	NO				NO				YES			
AIC	9698.56				9551.75				9699.11			
Obs. (N)	26873				26873				26873			

5.5 Frequency of treatment arms

Figure 2 compares the frequency of the different treatment arms across the different control variables. Overall, the figures show that all treatment arms include a large number of observations, even when stratified by province, device type, and weekday.

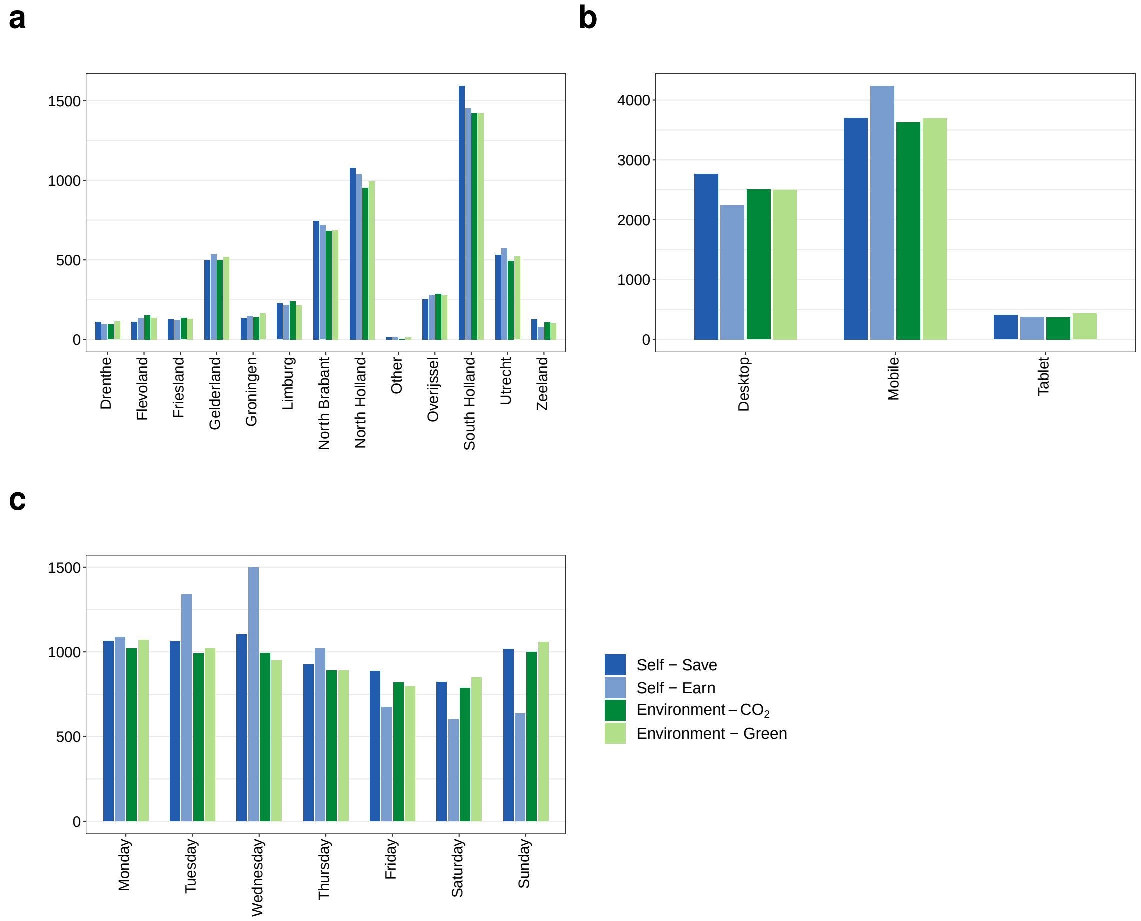

Supplementary Figure 2: Number of observations per condition for different control variables. Overall, $N = 26,873$ customers visited the e-commerce website during the 14-day period of the field experiment. **a**, Number of observations by conditions for the different provinces in the Netherlands from where a customer has accessed the website. **b**, Number of observations by condition for the device types a customer has accessed the website from. **c**, Number of observations by condition for the weekday on which customers have accessed the website.

6 Regional heterogeneity in the commitment to solar panels

We compared the treatment effect of message framing from the field experiment across different provinces in the Netherlands (Figure 3a–d). Importantly, we found that the intervention *Self–Save* generated the highest rate of commitment to solar panels in eight out of twelve provinces. However, the rate of commitment varied across provinces. For example, the highest rate of customers committing to solar panels for *Self–Save* (Figure 3a) was observed in South Holland (mean: 7.72 %) and Utrecht (mean: 6.94 %). In contrast, other regions such as the provinces Drenthe (mean: 2.70 %) and Overijssel (mean: 5.16 %), reported comparatively low rates of customers committing to solar panels for *Self–Save*.

There are several possible explanations for regional variations in the commitment to solar panels. For example, previous research found that the penetration rate of solar panels and peer effects are important drivers of solar panel installations [9, 28, 29, 30]. Hence, regions with a higher penetration rate (or where households have strong ties with neighbors) may also exhibit higher rates of commitment. Similarly, socioeconomic or demographic factors may explain heterogeneity in the adoption of solar panels [46], thus providing potential explanations for differences among provinces with distinct socioeconomic and demographic characteristics. As we conducted our experiment on an online website, heterogeneity in Internet use could potentially be relevant to the observed rate of commitment across different provinces. However, Internet penetration is high in the Netherlands with more than 90 % of the population being connected [47], making Internet use less likely to impact our results.

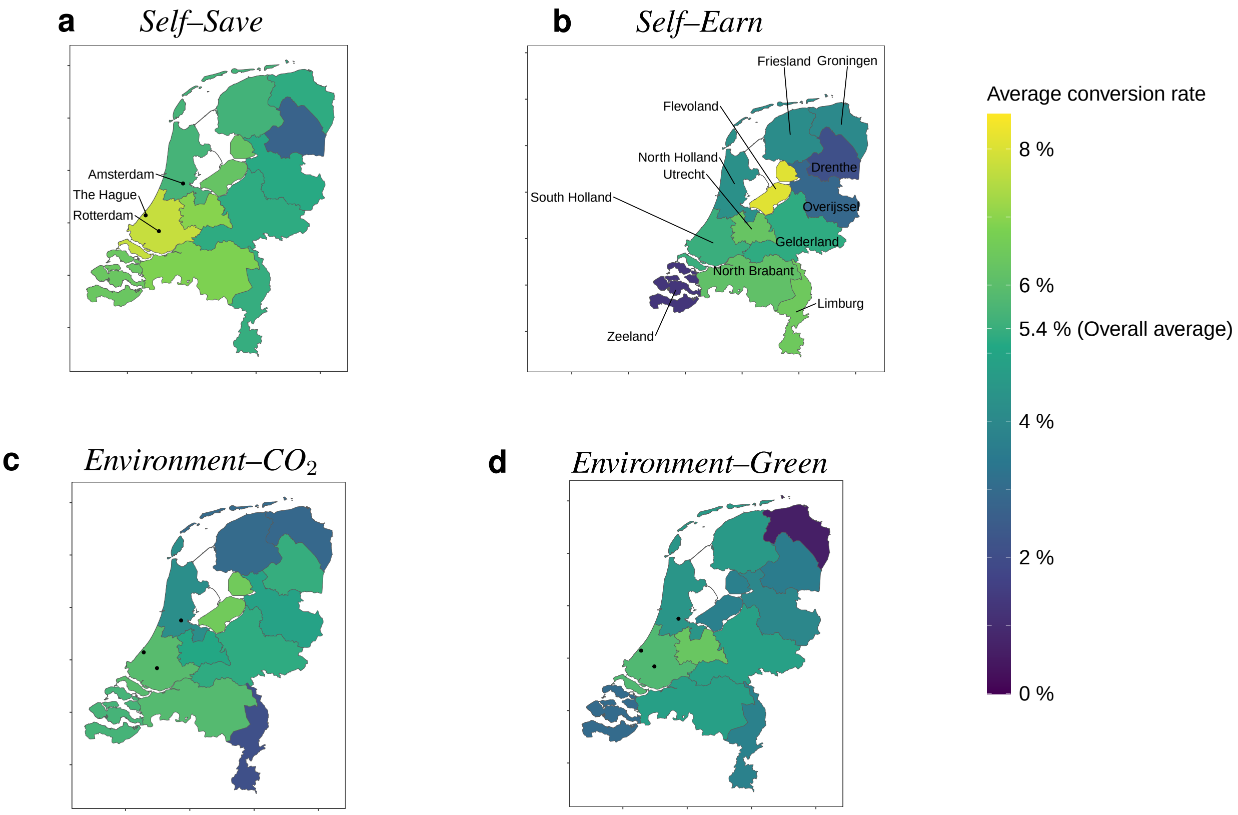

Supplementary Figure 3: Commitment to solar panels in the field experiment by province. The maps show the average rate of commitment among customers across provinces in the Netherlands. The plot further compares the four interventions: **a**, *Self–Save*; **b**, *Self–Earn*; **c**, *Environment–CO_2*; and **d**, *Environment–Green*. Overall, $N = 26,873$ customers visited the e-commerce website during the 14-day period of the field experiment.

Our analysis from above further suggests differences between urban regions such as South Holland, including the cities The Hague and Rotterdam, and Utrecht versus rural areas such as Drenthe and Overijssel. Urban regions are characterized by urban housing, high population density, and greater economic wealth which may positively affect the commitment to solar panels. However, previous research from the United States showed that the uptake of solar panels is negatively associated with the level of urbanization and the population density of an area [28, 46]. Hence, we study (1) how the commitment to solar panels varies between rural versus urban regions and (2) how population density affects customers' decisions to commit to solar panels.

To compare the commitment to solar panels across rural versus urban municipalities, we used the categorization from the Central Agency for Statistics (CBS), Netherlands, and encoded a municipality as urban ($= 1$; for the CBS labels “somewhat”, “heavily”, and “very heavily”) or rural ($= 0$; for the CBS labels “little urban“ and “not urban”). We then re-estimated the logistic model from the main analysis and included a binary variable for urbanization. The results are in Table 6. We find that the degree of urbanization has no statistically significant effect on the rate of customers committing to solar panels. Furthermore, the degree of urbanization does not affect the interventions’ effect on adoption behavior, as indicated by the non-significant interaction effects of these variables across common significance thresholds. Hence, we cannot argue that customers are committing to solar panels at larger rates in urban compared to rural environments.

We now compare how sparsely versus densely populated municipalities differ with respect to customers’ commitment behavior for solar panels. To do so, we include an additional variable in our regression model measuring the population density (i. e., inhabitants per km^2) in the municipality where a customer has accessed the website. The variable was standardized to mean zero and a standard deviation of one before the estimation. The results are in Table 7. A one standard deviation increase in population density does not lead to a significant increase in the mean rate of customers committing to solar panels across common significance levels. In sum, we do not find that regional differences explain the decision to commit to solar panels based on population density.

Supplementary Table 6: Treatment effect in rural versus urban municipalities. The regression analysis from the main paper is repeated with an additional control variable referring to the level of urbanization. The coding is urban $(= 1)$ and rural $(= 0)$. P-values are calculated using tow-sided t-tests. Abbreviations: b = coefficient, s.e. = standard error, CI = confidence interval, AIC = Akaike information criterion, Obs. (N) = Number of observations.

	Model 1				Model 2			
	b	$s.e.$	P	95 % CI	b	$s.e.$	P	95% CI
Self–Save	−2.74	0.07	0.00	[−2.88; −2.60]	−2.75	0.10	0.00	[−2.95; −2.56]
Self–Earn	−2.97	0.08	0.00	[−3.12; −2.83]	−2.92	0.11	0.00	[−3.14; −2.72]
Environment–CO_2	−2.98	0.08	0.00	[−3.14; −2.83]	−2.97	0.11	0.00	[−3.20; −2.76]
Environment–Green	−3.04	0.08	0.00	[−3.20; −2.89]	−3.10	0.12	0.00	[−3.33; −2.88]
Urban	0.08	0.07	0.21	[−0.04; 0.21]	0.08	0.07	0.20	[−0.04; 0.21]
Urban x (*Self–Earn*)					0.01	0.11	0.94	[−0.20; 0.22]
Urban x (*Environment–CO_2*)					−0.08	0.11	0.50	[−0.30; 0.15]
Urban x (*Environment–Green*)					−0.01	0.12	0.91	[−0.24; 0.22]
AIC	8833.80				8839.11			
Obs. (N)	21141				21141			

Supplementary Table 7: Treatment effect and impact of population density. Population density is measured in inhabitants per km^2. P-values are calculated using tow-sided t-tests. Abbreviations: b = coefficient, s.e. = standard error, CI = confidence interval, AIC = Akaike information criterion, Obs. (N) = Number of observations.

	Model 1				Model 2			
	b	$s.e.$	P	95 % CI	b	$s.e.$	P	95% CI
Self–Save	−2.68	0.06	0.00	[−2.80; −2.58]	−2.69	0.06	0.00	[−2.80; −2.58]
Self–Earn	−2.92	0.06	0.00	[−3.04; −2.80]	−2.92	0.06	0.00	[−3.04; −2.80]
Environment–CO_2	−2.93	0.06	0.00	[−3.05; −2.80]	−2.93	0.06	0.00	[−3.05; −2.80]
Environment–Green	−2.99	0.06	0.00	[−3.12; −2.86]	−2.99	0.07	0.00	[−3.12; −2.87]
Population density	0.03	0.03	0.35	[−0.03; 0.09]	0.03	0.03	0.40	[−0.03; 0.09]
Population density x (*Self–Earn*)					0.03	0.05	0.48	[−0.06; 0.13]
Population density x (*Environment–*CO_2)					−0.07	0.05	0.19	[−0.18; 0.03]
Population density x (*Environment–Green*)					−0.04	0.05	0.43	[−0.15; 0.06]
AIC	8834.51				8836.74			
Obs. (N)	21141				21141			

7 Online experiment

Experimental setup

In addition to the field experiment, we conducted a scenario-based controlled online experiment ($N = 1,000$) with two objectives: (1) We validate the results from the field experiment and (2) We check the comparability of our messages. The scenario-based online experiment was preregistered (https://osf.io/7fnr6) and approved by the Rotterdam School of Management, Internal Review Board (ETH2122-0288).

Our experiment follows a between-subjects design, which allows us to evaluate the separate effects of different informative cues on customers' adoption of solar panels. Overall, we test eight messages that are targeted at oneself or the environment where the outcome is presented as concrete or abstract (i. e., with or without numeric values).

For messages with a concrete outcome, we test the following four conditions: (1) *Self–Save*–Concrete: "Save on average € 813 per year," (2) *Self–Earn*–Concrete: "Earn on average € 813 per year," (3) *Environment–*CO_2–Concrete: "Reduce your emissions on average by 2.0 t CO2 per year," and (4) *Environment–Green*–Concrete: "Generate on average 2.8 MWh green electricity per year."

For messages with abstract outcomes, we test the following four conditions: (1) *Self–Save*–Abstract: "Save money," (2) *Self–Earn*–Abstract: "Earn money," (3) *Environment–*CO_2–Abstract: "Reduce CO2 emissions," and (4) *Environment–Green*–Abstract: "Generate green electricity."

Consistent with the field experiment, the messages refer to average financial savings of EUR 813 per year. Furthermore, we report an environmental impact of 2.0 t CO_2 and an electricity generation of 2.8 MWh per year. These values correspond to the average CO_2 emissions and electricity consumption of a typical household in the Netherlands in 2021 [42, 43, 48]. They represent the potential emission reduction or green electricity production achievable by installing solar panels that can cover the household's annual electricity consumption.

To support our choice behind the messages in the field experiment, we specifically test two abstract messages targeting oneself without numeric values (i. e., *Self–Save*–Abstract, *Self–Earn*–Abstract) and two concrete messages targeting the environment with numeric values (i. e., *Environment–*CO_2–Concrete, *Environment–Green*–Concrete).

We expect that concrete messages presenting monetary outcomes with numeric values will be more impactful compared to abstract messages without numeric values, as individuals can easily relate to them. We did not expect that messages targeting the environment would necessarily be more or less effective when displayed as concrete quantifications of the environmental impact, as it is generally difficult for individuals to relate to numerical values in this context [49,50]. Moreover, we expect that messages targeted at oneself would result in the highest adoption rate, which, in turn, provides empirical support for the choice of our messages in the field experiment.

At the beginning of the online experiment, participants were given instructions to imagine a situation in which they are a homeowner who is considering to install solar panels (see Section 7.1 for the instructions). This is consistent with our field experiment where the online retailer advertises PV systems using the term "solar panels" given its wide use among potential customers. Additionally, participants were explicitly instructed to disregard their personal financial and housing situations. All participants provided informed consent.

The participants were then introduced to a fictitious e-commerce website presenting solar panels for purchase. The presentation was similar to that of the field experiment and was shown to all participants in English (and thus we only recruited participants fluent in English). The interface included (i) a picture of solar panels installed on a rooftop, (ii) information describing the installation process, (iii) a dummy button labeled "Buy Now" to purchase the solar panels, and (iv) a call-to-action box advertising the solar panels. Here, (iv) is important because the text varied according to the intervention. The different experimental conditions are shown in Figure 4.

Using a between-group design, the participants were then asked to rate the likelihood of purchasing solar panels on this website on a Likert scale from 1 (= very unlikely) to 7 (= very likely).

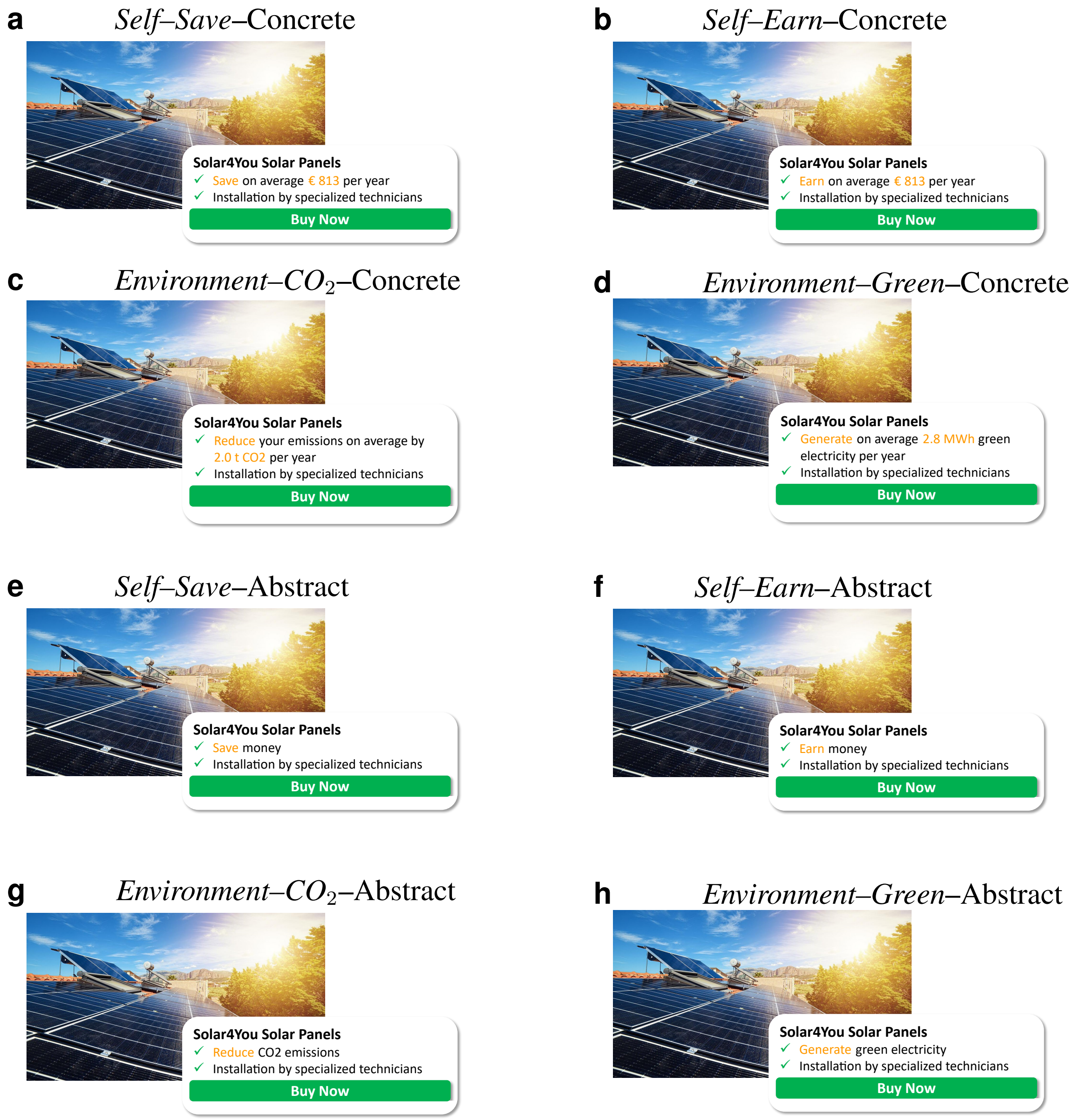

Supplementary Figure 4: Interface of online experiment with different messages. The interface shows a fictional e-commerce website offering solar panels. Using message framing, the presentation is varied according to the different messages: **a**, *Self–Save*–Concrete, **b**, *Self–Earn*–Concrete, **c**, *Environment–*CO_2–Concrete, **d**, *Environment–Green*–Concrete, **e**, *Self–Save*–Abstract, **f**, *Self–Earn*–Abstract, **g**, *Environment–*CO_2–Abstract, and **h**, *Environment–Green*–Abstract. Keywords referring to the target (i. e., oneself or the environment) and the framing in the presentation were additionally highlighted in orange color during the online experiment.

Study population

We recruited $N = 1000$ participants (mean age $= 50.93$ years; 54.00 % women) fluent in English from the Netherlands via MSI (https://www.msi-aci.com/). However, the majority of the Dutch population (i.e., around 90 %) are fluent in English [51]. We randomly assigned participants to one of the eight interventions. During the experiment, participants were not bound to any time constraints.

Participants who fail our attention checks are excluded from the analysis. We implemented two attention checks (see Table 9) during the experiment: The first attention check appears before the experiment. The second attention check appears after the main experiment. The exact questions for both attention checks are in Section 7.1.

Statistical analysis

To estimate the effect of the interventions, we followed the statistical analysis from the field experiment. Consistent with our preregistration, the dependent variable was constructed by mapping the Likert scale with the likelihood of adoption onto a dichotomous outcome (i. e., whether someone adopted solar panels yes/no). To do so, a participant is classified as having purchased (not purchased) solar panels if the participant indicated the likelihood of purchasing solar panels with a Likert rating of 5 or higher (4 or below). This dichotomization ensures that the estimates are comparable with those from the field where the dichotomous outcomes were collected. Subsequently, we estimated a logistic regression analogous to that of the field experiment.

The adoption rate for each intervention is shown in Figure 5a. We find that the treatment effect varies across the different interventions. Consistent with our results from the field experiment, concrete messages targeting oneself result in the highest adoption rates for solar panels. Here, 48.41 % (44.92 %) participants express an intention to purchase solar panels when highlighting additional earnings (cost savings) for oneself. In contrast, abstract messages targeting oneself

(i. e., without numeric values) and messages targeting the environment led to lower adoption rates. Specifically, only 38.58 % of the participants in the *Self–Save*–Abstract condition, 41.67 % in the *Self–Earn*–Abstract condition, 34.72 % in the *Environment–CO_2*–Concrete condition, 27.20 % in the *Environment–Green*–Concrete condition, 44.88 % in the *Environment–CO_2*–Abstract condition, and 31.86 % in the *Environment–Green*–Abstract condition expressed an intention to adopt solar panels. Importantly, abstract messages without numeric values result in higher adoption rates for solar panels when targeting the environment thus supporting the choice of our messages in the field experiment.

In the next step, we performed regression analysis to estimate the treatment effect and thus make statistical comparisons across the conditions. The estimation results showing the mean adoption rate by condition are in Figure 5b and Table 8. We report both the standard error (SE) and 95 % confidence interval (CI) in the following. Framing solar panel adoption as additional earnings for oneself has the largest estimated coefficient (coef: -0.06, SE $= 0.18$, $t = -0.36$, $P = 0.722$, 95 % CI $= [-0.41, 0.29]$). This corresponds to an estimated rate of customers expressing an intent to adopt solar panels of 48.41 % (i. e., after applying the transformation $e^{\alpha_{\text{Condition[i]}}}/(1 + e^{\alpha_{\text{Condition[i]}}})$). For the other interventions, we find smaller coefficients. In particular, this is the case for *Self–Save*–Concrete (coef: -0.20, SE $= 0.19$, $t = -1.10$, $P = 0.270$, 95 % CI $= [-0.57, 0.16]$), *Self–Save*–Abstract (coef: -0.46, SE $= 0.18$, $t = -2.55$, $P = 0.11$, 95 % CI $= [-0.83, -0.11]$), *Self–Earn*–Abstract (coef: -0.34, SE $= 0.19$, $t = -1.82$, $P = 0.069$, 95 % CI $= [-0.70, 0.02]$), *Environment–CO_2*–Concrete (coef: -0.63, SE $= 0.18$, $t = -3.61$, $P < 0.001$, 95 % CI $= [-0.98, -0.29]$), *Environment–Green*–Concrete (coef: -0.98, SE $= 0.20$, $t = -4.90$, $P < 0.001$, 95 % CI $= [-1.39, -0.60]$), *Environment–CO_2*–Abstract (coef: -0.21, SE $= 0.18$, $t = -1.15$, $P = 0.250$, 95 % CI $= [-0.56, 0.14]$), and *Environment–Green*–Abstract (coef: -0.76, SE $= 0.20$, $t = -3.77$, $P < 0.001$, 95 % CI $= [-1.17, -0.37]$).

For all coefficients, the 95 % CIs overlap (except for *Self–Save*–Concrete and *Self–Earn*–Concrete, which are significantly larger compared to *Environment–Green*–Concrete) and thus no

ranking can be made for the online experiment. The wide CIs may be a result of the sample size in the online experiment compared to the field experiment such that the size of the estimated coefficients may still indicate which interventions would be particularly effective in the field.

The results of the online experiment suggest that message framing targeting oneself tends to result in higher adoption rates for solar panels compared to messages targeting the environment regardless of whether the outcome is presented as concrete or abstract (i. e., with or without numeric values). Furthermore, abstract messages tend to result in higher adoption rates for solar panels when targeting the environment. Overall, the results of the online experiment thus support the choice of our messages in the field experiment.

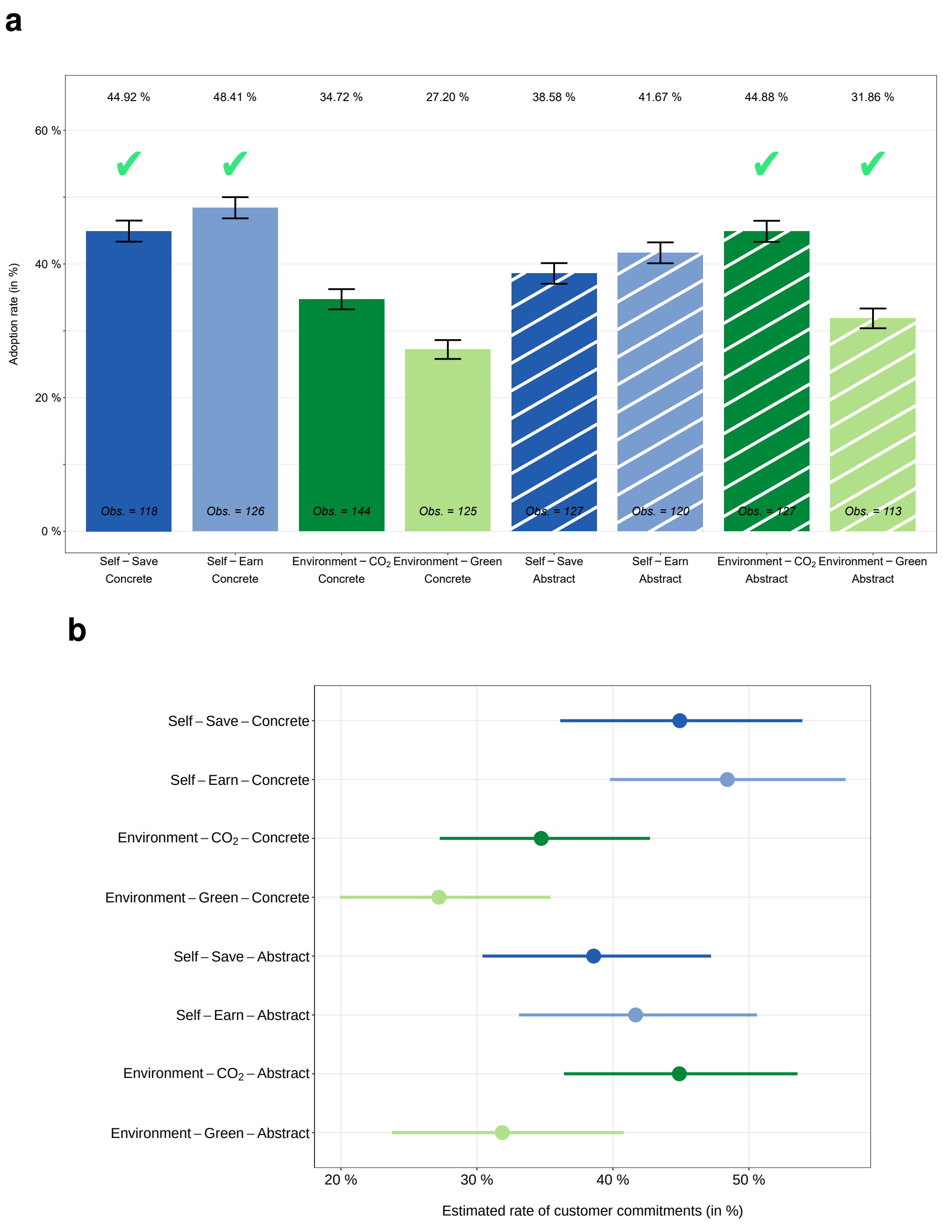

Supplementary Figure 5: Effect of message framing on the adoption of solar panels in the online experiment. **a**, Observed rate of customers deciding to adopt solar panels. Here, the observations (abbreviated by "Obs.") are the number of participants per treatment arm. Overall, we recruited $N = 1,000$ participants. Messages that resulted in the largest number of participants expressing an intent to adopt solar panels were included in the field experiment (shown by green check marks ✓). We find that concrete messages targeting oneself result in the highest adoption rate for solar panels. Importantly, we find that abstract messages result in a higher adoption rate for solar panels when targeting the environment thus supporting the choice of our messages in the field experiment. **b**, Estimated effect of the different interventions on the adoption rate using the regression model. The coefficients capture the effect of each intervention on the decision to adopt solar panels $(= 1)$ or not $(= 0)$. For all coefficients, the 95 % CIs overlap (except for *Self–Save*–Concrete and *Self–Earn*–Concrete, which are significantly larger compared to *Environment–Green*–Concrete) and thus no ranking can be made for the online experiment. However, the size of the estimated rate of customer commitments may still indicate which interventions would be particularly effective in the field. Overall, we recruited $N = 1,000$ participants. Shown are the estimated coefficients as the mean rate of customers expressing an intent to adopt solar panels (i. e., by transforming the original coefficient $\alpha_{\text{Condition[i]}}$ via $e^{\alpha_{\text{Condition[i]}}}/(1 + e^{\alpha_{\text{Condition[i]}}})$) (dot) and 95 % CI (bars).

Supplementary Table 8: Estimation results for the statistical analysis of the online experiment. The table shows the regression results for our logistic regression for the online experiment. The first column reports the estimated coefficients on a log-odds scale ($\alpha_{\text{Condition[i]}}$) from the logistic regression. The last column (Rate of commitment) reports the corresponding estimated rate of commitment (i. e., $\mathrm{e}^{\alpha_{\text{Condition[i]}}}/(1+\mathrm{e}^{\alpha_{\text{Condition[i]}}})$). Note that coefficients may be negative on the log-odds scale, i. e., prior to applying the inverse-logit transformation. P-values are calculated using tow-sided t-tests. Abbreviations: b = coefficient, s.e. = standard error, CI = confidence interval, AIC = Akaike information criterion, Obs. (N) = Number of observations.

	b	$s.e.$	P	95 % CI	Rate of commitment
Self–Save–Concrete	−0.20	0.19	0.27	[−0.57; 0.16]	0.45
Self–Earn–Concrete	−0.06	0.18	0.72	[−0.41; 0.29]	0.48
Environment–CO_2–Concrete	−0.63	0.18	0.00	[−0.98; −0.29]	0.35
Environment–Green–Concrete	−0.98	0.20	0.00	[−1.39; −0.60]	0.27
Self–Save–Abstract	−0.46	0.18	0.01	[−0.83; −0.11]	0.39
Self–Earn–Abstract	−0.34	0.19	0.07	[−0.70; 0.02]	0.42
Environment–CO_2–Abstract	−0.21	0.18	0.25	[−0.56; 0.14]	0.45
Environment–Green–Abstract	−0.76	0.20	0.00	[−1.17; −0.37]	0.32
AIC	1333.72				
Obs. (N)	1000				

7.1 Survey for online experiment

Instructions

Participants in the scenario-based online experiment were presented with the following instructions before beginning the experiment. First, we asked them to imagine the following situation:

> "Imagine the following situation: You are a house owner and think about installing a solar panel on your roof. You already know that your house is suitable for installation and discuss the topic with a friend. At some point, she/he is referring you to a new website "Solar4You." The website offers solar panel installations for purchase. In the experiment, you will see the website."

Subsequently, the participants were asked to perform the following task:

> "We will show you a screenshot of the "Solar4You" website. Your task is to rate the likelihood that you will purchase solar panels based on what you see. When answering the following questions, please do not take into account your own financial or housing situation. Your decision should be solely based on the presentation."

Survey questions

Table 9 lists the survey questions for the dependent variable, attention checks, and manipulation checks for the scenario-based online experiment.

Supplementary Table 9: Survey questions for the scenario-based online experiment.

Question	Answers
Dependent variable:	
Based on the presentation above, what is the likelihood that you will purchase the solar panel?	[Very unlikely (1) / 2 /. . . / 6 / Very likely (7)]
Attention check 1 (before the experiment):	
Participants in surveys have a wide variety of interests and participate in a wide variety of sports. We appreciate your help with this survey and are interested in whether you take enough time to read the survey directions and questions carefully before you provide your answers. In order to demonstrate that you have read these instructions carefully, choose the "I do not play any sports" answer. Thank you for your cooperation and participation in this study.	[Soccer / Tennis / Rugby / I do not play any sports]
Attention check 2 (after the experiment):	
Please answer the following question to show that you have read the website carefully:	
• What is the main reason for buying solar panels at Solar4You?	[Delivery is for free / High quality of solar panels / Installation by specialized technicians]

References

[1] Gupta, S. & Ogden, D. T. To buy or not to buy? A social dilemma perspective on green buying. *Journal of Consumer Marketing* **26**, 376–391 (2009).

[2] Evans, L. *et al.* Self-interest and pro-environmental behaviour. *Nature Climate Change* **3**, 122–125 (2013).

[3] Bolderdijk, J. W., Steg, L., Geller, E. S., Lehman, P. K. & Postmes, T. Comparing the effectiveness of monetary versus moral motives in environmental campaigning. *Nature Climate Change* **3**, 413–416 (2013).

[4] Segev, S., Fernandes, J. & Wang, W. The Effects of gain versus loss message framing and point of reference on consumer responses to green advertising. *Journal of Current Issues & Research in Advertising* **36**, 35–51 (2015).

[5] White, K., Habib, R. & Hardisty, D. J. How to SHIFT Consumer Behaviors to be More Sustainable: A Literature Review and Guiding Framework. *Journal of Marketing* **83**, 22–49 (2019).

[6] Grazzini, L., Rodrigo, P., Aiello, G. & Viglia, G. Loss or gain? The role of message framing in hotel guests' recycling behaviour. *Journal of Sustainable Tourism* **26**, 1944–1966 (2018).

[7] Gustafson, A. *et al.* The durable, bipartisan effects of emphasizing the cost savings of renewable energy. *Nature Energy* **7**, 1023–1030 (2022).

[8] Kastner, I. & Stern, P. C. Examining the decision-making processes behind household energy investments: A review. *Energy Research & Social Science* **10**, 72–89 (2015).

[9] Rai, V., Reeves, D. C. & Margolis, R. Overcoming barriers and uncertainties in the adoption of residential solar PV. *Renewable Energy* **89**, 498–505 (2016).

[10] Stern, P. C., Wittenberg, I., Wolske, K. S. & Kastner, I. Household production of photovoltaic energy. In Lewis, A. (ed.) *The Cambridge Handbook of Psychology and Economic Behaviour*, 541–566 (Cambridge University Press, Cambridge, UK, 2018).

[11] van der Linden, S. Warm glow is associated with low- but not high-cost sustainable behaviour. *Nature Sustainability* **1**, 28–30 (2018).

[12] Mazar, N., Amir, O. & Ariely, D. The dishonesty of honest people: A theory of self-concept maintenance. *Journal of Marketing Research* **45**, 633–644 (2008).

[13] Ariely, D., Bracha, A. & Meier, S. Doing good or doing well? Image motivation and monetary incentives in behaving prosocially. *American Economic Review* **99**, 544–555 (2009).

[14] Pew Research Center. The politics of climate change: Everday environmentalism (2016). URL https://www.pewresearch.org/science/2016/10/04/everyday-environmentalism/.

[15] European Commission. Special Eurobarometer 513: Climate change (2021). URL https://europa.eu/eurobarometer/surveys/detail/2273.

[16] Karp, D. G. Values and their effect on pro-environmental behavior. *Environment and Behavior* **28**, 111–133 (1996).

[17] Minton, A. P. & Rose, R. L. The Effects of Environmental Concern on Environmentally Friendly Consumer Behavior: An Exploratory Study. *Journal of Business Research* **40**, 37–48 (1997).

[18] Poortinga, W., Steg, L. & Vlek, C. Values, environmental concern, and environmental behavior. *Environment and Behavior* **36**, 70–93 (2004).

[19] Asensio, O. I. & Delmas, M. A. Nonprice incentives and energy conservation. *PNAS* **112**, E510–E515 (2015).

[20] Schwirplies, C. & Ziegler, A. Offset carbon emissions or pay a price premium for avoiding them? A cross-country analysis of motives for climate protection activities. *Applied Economics* **48**, 746–758 (2016).

[21] Tiefenbeck, V. *et al.* Overcoming Salience Bias: How Real-Time Feedback Fosters Resource Conservation. *Management Science* **64**, 1458–1476 (2018).

[22] Nisa, C. F., Bélanger, J. J., Schumpe, B. M. & Faller, D. G. Meta-analysis of randomised controlled trials testing behavioural interventions to promote household action on climate change. *Nature Communications* **10**, 4545 (2019).

[23] Schwartz, D., Loewenstein, G. & Agüero-Gaete, L. Encouraging pro-environmental behaviour through green identity labelling. *Nature Sustainability* **3**, 746–752 (2020).

[24] Steg, L. Psychology of climate change. *Annual Review of Psychology* **74**, 391–421 (2023).

[25] Allcott, H. Social norms and energy conservation. *Journal of Public Economics* **95**, 1082–1095 (2011).

[26] Allcott, H. & Rogers, T. The Short-Run and Long-Run Effects of Behavioral Interventions: Experimental Evidence from Energy Conservation. *American Economic Review* **104**, 3003–3037 (2014).

[27] Bonan, J., Cattaneo, C., d'Adda, G. & Tavoni, M. The interaction of descriptive and injunctive social norms in promoting energy conservation. *Nature Energy* **5**, 900–909 (2020).

[28] Bollinger, B. & Gillingham, K. Peer Effects in the Diffusion of Solar Photovoltaic Panels. *Marketing Science* **31**, 900–912 (2012).

[29] Graziano, M. & Gillingham, K. Spatial patterns of solar photovoltaic system adoption: The influence of neighbors and the built environment. *Journal of Economic Geography* **15**, 815–839 (2015).

[30] Wolske, K. S., Stern, P. C. & Dietz, T. Explaining interest in adopting residential solar photovoltaic systems in the United States: Toward an integration of behavioral theories. *Energy Research & Social Science* **25**, 134–151 (2017).

[31] Herberz, M., Hahnel, U. J. J. & Brosch, T. Counteracting electric vehicle range concern with a scalable behavioural intervention. *Nature Energy* **7**, 503–510 (2022).

[32] Lindenberg, S. Prosocial behavior, solidarity, and framing processes. In Fetchenhauer, D., Flache, A., Buunk, B. & Lindenberg, S. (eds.) *Solidarity and prosocial behavior: An integration of sociological and psychological perspectives*, 23–44 (Springer, Berlin, Germany, 2006).

[33] Lindenberg, S. Social rationality, semi-modularity and goal-framing: What is it all about? *Analyse & Kritik* **30**, 669–687 (2008).

[34] Lindenberg, S. & Steg, L. Normative, Gain and Hedonic Goal Frames Guiding Environmental Behavior. *Journal of Social Issues* **63**, 117–137 (2007).

[35] Bargh, J. A., Gollwitzer, P. M., Lee-Chai, A., Barndollar, K. & Trötschel, R. The automated will: Nonconscious activation and pursuit of behavioral goals. *Journal of Personality and Social Psychology* **81**, 1014–1027 (2001).

[36] Centraal Bureau voor de Statistiek. Renewable electricity; production and capacity (2023). URL https://opendata.cbs.nl/#/CBS/en/dataset/82610ENG/table?ts=1686733248675.

[37] SolarPower Europe. EU market outlook for solar power 2022 - 2026 (2022). URL https://www.solarpowereurope.org/insights/market-outlooks/eu-market-outlook-for-solar-power-2022-2026-2.

[38] U.S. Energy Information Administration. Wind, solar, and batteries increasingly account for more new U.S. power capacity additions (2023). URL

https://www.eia.gov/todayinenergy/detail.php?id=55719#:~:text=As%20the%20cost%20of%20solar,6%25%20of%20the%20U.S.%20total.

[39] Centraal Bureau voor de Statistiek. Three-quarters of Dutch concerned about impact of climate change (2021). URL https://www.cbs.nl/en-gb/news/2021/22/three-quarters-of-dutch-concerned-about-impact-of-climate-change.

[40] International Energy Agency. The global energy crisis (2022). URL https://www.iea.org/reports/world-energy-outlook-2022/the-global-energy-crisis.

[41] Netherlands Enterprise Agency. Subsidy for sustainable energy and energy saving for owner-occupied homes (ISDE) (2023). URL https://www.rvo.nl/subsidies-financiering/isde/woningeigenaren.

[42] Centraal Bureau voor de Statistiek. Average energy prices for consumers (2023). URL https://www.cbs.nl/en-gb/figures/detail/84672ENG#.

[43] Centraal Bureau voor de Statistiek. Energy consumption private dwellings; type of dwelling and regions (2023). URL https://www.cbs.nl/en-gb/figures/detail/81528ENG?q=parts%20of%20the%20country.

[44] TU Delft. Detailed PV system design (2023). URL https://www.tudelft.nl/en/ewi/over-de-faculteit/afdelingen/electrical-sustainable-energy/photovoltaic-materials-and-devices/dutch-pv-portal/tools-models/detailed-pv-system-design.

[45] Wirth, H. Recent facts about photovoltaics in Germany (2023). URL https://www.ise.fraunhofer.de/en/publications/studies/recent-facts-about-pv-in-germany.html.

[46] Lukanov, B. R. & Krieger, E. M. Distributed solar and environmental justice: Exploring the demographic and socio-economic trends of residential PV adoption in California. *Energy Policy* **134**, 110935 (2019).

[47] The World Bank. Individuals using the Internet (% of population) - Netherlands (2023). URL https://data.worldbank.org/indicator/IT.NET.USER.ZS?locations=NL.

[48] United States Environmental Protection Agency. Greenhouse gas equivalencies calculator (2021). URL https://www.epa.gov/energy/greenhouse-gas-equivalencies-calculator.

[49] Bleys, B., Defloor, B., van Ootegem, L. & Verhofstadt, E. The environmental impact of individual behavior: Self-assessment versus the ecological footprint. *Environment and Behavior* **50**, 187–212 (2018).

[50] Dechezleprêtre, A. *et al.* Fighting climate change: International attitudes toward climate policies. *NBER Working Paper Series* 30265 (2022).

[51] European Commission. Europeans and their languages (2012). URL https://europa.eu/eurobarometer/surveys/detail/1049.
