# Supplementary material for: Message framing to promote solar panels
Source: Nat Commun. 2023 Nov 8;14:7187. doi: 10.1038/s41467-023-42904-0 (PMC10632465; doi:10.1038/s41467-023-42904-0)
Supplement: Supplementary file 3 — Reporting Summary [file 41467_2023_42904_MOESM3_ESM.pdf]

nature portfolio

Corresponding author(s): Stefan Feuerriegel

Last updated by author(s): Oct 9, 2023

Reporting Summary

Nature Portfolio wishes to improve the reproducibility of the work that we publish. This form provides structure for consistency and transparency in reporting. For further information on Nature Portfolio policies, see our Editorial Policies and the Editorial Policy Checklist.

Statistics

For all statistical analyses, confirm that the following items are present in the figure legend, table legend, main text, or Methods section.

n/a	Confirmed	
☐	☒	The exact sample size (*n*) for each experimental group/condition, given as a discrete number and unit of measurement
☐	☒	A statement on whether measurements were taken from distinct samples or whether the same sample was measured repeatedly
☐	☒	The statistical test(s) used AND whether they are one- or two-sided<br>*Only common tests should be described solely by name; describe more complex techniques in the Methods section.*
☐	☒	A description of all covariates tested
☐	☒	A description of any assumptions or corrections, such as tests of normality and adjustment for multiple comparisons
☐	☒	A full description of the statistical parameters including central tendency (e.g. means) or other basic estimates (e.g. regression coefficient) AND variation (e.g. standard deviation) or associated estimates of uncertainty (e.g. confidence intervals)
☐	☒	For null hypothesis testing, the test statistic (e.g. *F*, *t*, *r*) with confidence intervals, effect sizes, degrees of freedom and *P* value noted<br>*Give P values as exact values whenever suitable.*
☒	☐	For Bayesian analysis, information on the choice of priors and Markov chain Monte Carlo settings
☒	☐	For hierarchical and complex designs, identification of the appropriate level for tests and full reporting of outcomes
☒	☐	Estimates of effect sizes (e.g. Cohen's *d*, Pearson's *r*), indicating how they were calculated

Our web collection on statistics for biologists contains articles on many of the points above.

Software and code

Policy information about availability of computer code

Data collection	No software was used.
Data analysis	The statistical analysis was implemented in R 4.2.2 using the packages "stats" (4.2.2) and "lme4" (1.1.31). Code for the data analysis can be viewed via OSF (https://osf.io/up74v/)

For manuscripts utilizing custom algorithms or software that are central to the research but not yet described in published literature, software must be made available to editors and reviewers. We strongly encourage code deposition in a community repository (e.g. GitHub). See the Nature Portfolio guidelines for submitting code & software for further information.

Data

Policy information about availability of data

All manuscripts must include a data availability statement. This statement should provide the following information, where applicable:

- Accession codes, unique identifiers, or web links for publicly available datasets
- A description of any restrictions on data availability
- For clinical datasets or third party data, please ensure that the statement adheres to our policy

All data from the online experiment is available via OSF (https://osf.io/7fnr6). Because of a non-disclosure agreement, access to data for the field experiment requires authorization from the partner company. Data access can be requested via Ting Li (tli@rsm.nl). Data use agreements are subject to our partner company's availability and internal regulations.

Research involving human participants, their data, or biological material

Policy information about studies with human participants or human data. See also policy information about sex, gender (identity/presentation), and sexual orientation and race, ethnicity and racism.

Reporting on sex and gender	Main study: not applicable, we did not have access to any sociodemographic variables. Additional analysis: Self-reported gender: Woman = 54.00%, Man = 45.90 %, Other = 0.10%; All participants provided informed consent.
Reporting on race, ethnicity, or other socially relevant groupings	Main study: not applicable, we did not have access to any sociodemographic variables. Additional analysis: Self-reported gender: Woman = 54.00%, Man = 45.90 %, Other = 0.10%
Population characteristics	See above.
Recruitment	Main study: Participants were all visitors to the website. Additional analysis: Participants were recruited via MSI.
Ethics oversight	Main study: Rotterdam School of Management, Internal Review Board. Additional analysis: Rotterdam School of Management, Internal Review Board.

Note that full information on the approval of the study protocol must also be provided in the manuscript.

Field-specific reporting

Please select the one below that is the best fit for your research. If you are not sure, read the appropriate sections before making your selection.

☐ Life sciences ☒ Behavioural & social sciences ☐ Ecological, evolutionary & environmental sciences

For a reference copy of the document with all sections, see nature.com/documents/nr-reporting-summary-flat.pdf

Behavioural & social sciences study design

All studies must disclose on these points even when the disclosure is negative.

Study description	Following a field and online experiment, a quantitative analysis was conducted to analyze the effect of message framing on solar panel adoptions.
Research sample	Main study (field experiment): all visitors to the website; we did not have access to any sociodemographic variables. Additional analysis: Online experiment with participants recruited via MSI. Participants were from the Netherlands and fluent in English to ensure comparability with the field experiment. Woman = 54.00%, Man = 45.90 %, Other = 0.01%; All participants provided informed consent.
Sampling strategy	Main study: All visitors of the website were included in the sample and randomly assigned to a treatment condition. The size was determined by the number of visitors to the website during the study period. Additional analysis: random sampling. The N was preregistered and was defined analogous to other online experiments (e.g., Herberz et al. 2022).
Data collection	Main study: the data was directly recorded by the partner company and then shipped to us. Additional analysis: this was a standard online experiment with participants recruited via MSI. In both studies, no specialized equipment was used and researchers were blinded to experimental conditions.
Timing	Main study: the time frame was March 22, 2021 through April 5, 2021. Additional analysis: Data collection until we reached our preregistered sample size of 1000; Start date: June 6, 2023 Stop date: July 11, 2023
Data exclusions	Main study: none. Additional analysis: two attention checks as preregistered.
Non-participation	Main study: all visitors were included with no non-participation. Online experiment: no participants dropped out of the study.
Randomization	Random allocation to treatment arms

Reporting for specific materials, systems and methods

We require information from authors about some types of materials, experimental systems and methods used in many studies. Here, indicate whether each material, system or method listed is relevant to your study. If you are not sure if a list item applies to your research, read the appropriate section before selecting a response.

Materials & experimental systems

n/a	Involved in the study	
☒	☐	Antibodies
☒	☐	Eukaryotic cell lines
☒	☐	Palaeontology and archaeology
☒	☐	Animals and other organisms
☒	☐	Clinical data
☒	☐	Dual use research of concern
☒	☐	Plants

Methods

n/a	Involved in the study	
☒	☐	ChIP-seq
☒	☐	Flow cytometry
☒	☐	MRI-based neuroimaging
